# (*E*)-*N*′-(4-Fluoro­benzyl­idene)-2-(3-methyl­phen­yl)acetohydrazide

**DOI:** 10.1107/S1600536813004467

**Published:** 2013-02-23

**Authors:** A. S. Praveen, Jerry P. Jasinski, Amanda C. Keeley, H. S. Yathirajan, B. Narayana

**Affiliations:** a Department of Studies in Chemistry, University of Mysore, Manasagangotri, Mysore 570 006, India; b Department of Chemistry, Keene State College, 229 Main Street, Keene, NH 03435-2001, USA; c Department of Studies in Chemistry, Mangalore University, Mangalagangotri, 574 199, India

## Abstract

In the title compound, C_16_H_15_FN_2_O, the dihedral angles between the benzene rings are74.7(8), 74.1 (1), 74.2 (7) and 74.3 (5)° in the four independent mol­ecules in the asymmetric unit. In the crystal, N—H—O hydrogen bonds involving the hydrazide and acetyl groups, which form *R*_2_^2^(18) ring motifs, link the mol­ecules into dimers, which form columns along [010].

## Related literature

For Schiff bases as ligands for complexation of metal ions, see: Aydogan *et al.* (2001 ▶); their applications as dyes and pigments, see: Taggi *et al.* (2002 ▶) and crystallography and coordination chemistry, see: Kundu *et al.* (2005 ▶); Xu *et al.* (1997 ▶). For related structures, see: Fun *et al.* (2011*a* ▶,*b* ▶, 2012 ▶); He & Shi (2011 ▶); Odabaşoğlu *et al.* (2007 ▶). For standard bond lengths, see: Allen *et al.* (1987 ▶).
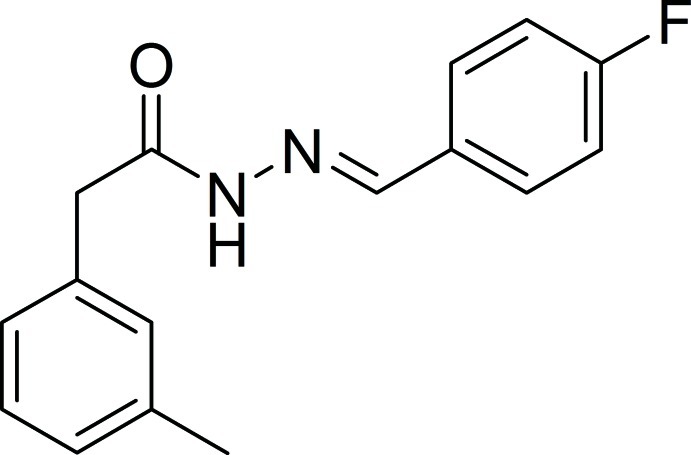


## Experimental

### 

#### Crystal data

C_16_H_15_FN_2_O
*M* *_r_* = 270.30
Triclinic, 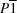

*a* = 11.8535 (7) Å
*b* = 12.3769 (9) Å
*c* = 20.8721 (11) Å
α = 98.549 (5)°
β = 103.074 (5)°
γ = 105.134 (6)°
*V* = 2808.2 (3) Å^3^
*Z* = 8
Cu *K*α radiation
μ = 0.74 mm^−1^
*T* = 173 K
0.22 × 0.16 × 0.08 mm

#### Data collection

Agilent Xcalibur (Eos, Gemini) diffractometer
Absorption correction: multi-scan (*CrysAlis PRO* and *CrysAlis RED*; Agilent, 2012 ▶) *T*_min_ = 0.780, *T*_max_ = 1.000
16410 measured reflections
9250 independent reflections
3807 reflections with *I* > 2σ(*I*)
*R*_int_ = 0.027

#### Refinement

*R*[*F* ^2^ > 2σ(*F* ^2^)] = 0.051
*wR*(*F* ^2^) = 0.191
*S* = 0.99
9250 reflections
725 parameters
H-atom parameters constrained
Δρ_max_ = 0.19 e Å^−3^
Δρ_min_ = −0.22 e Å^−3^

### 

Data collection: *CrysAlis PRO* (Agilent, 2012 ▶); cell refinement: *CrysAlis PRO*; data reduction: *CrysAlis RED* (Agilent, 2012 ▶); program(s) used to solve structure: *SHELXS97* (Sheldrick, 2008 ▶); program(s) used to refine structure: *SHELXL97* (Sheldrick, 2008 ▶); molecular graphics: *SHELXTL* (Sheldrick, 2008 ▶); software used to prepare material for publication: *SHELXTL* and *Mercury* (Macrae *et al.*, 2008 ▶).

## Supplementary Material

Crystal structure: contains datablock(s) global, I. DOI: 10.1107/S1600536813004467/lx2277sup1.cif

Structure factors: contains datablock(s) I. DOI: 10.1107/S1600536813004467/lx2277Isup2.hkl

Supplementary material file. DOI: 10.1107/S1600536813004467/lx2277Isup3.cml

Additional supplementary materials:  crystallographic information; 3D view; checkCIF report

## Figures and Tables

**Table 1:** Hydrogen-bond geometry (Å, °)

*D*—H⋯*A*	*D*—H	H⋯*A*	*D*⋯*A*	*D*—H⋯*A*
N1*A*—H1*A*⋯O1*B* ^i^	0.86	2.01	2.868 (3)	173
N1*B*—H1*B*⋯O1*A* ^i^	0.86	2.00	2.860 (3)	173
N1*C*—H1*C*⋯O1*D* ^ii^	0.86	2.01	2.865 (3)	173
N1*D*—H1*D*⋯O1*C* ^iii^	0.86	2.00	2.857 (4)	173

Symmetry codes: (i) 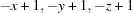; (ii) 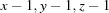; (iii) 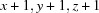.

**Fig. 1.**
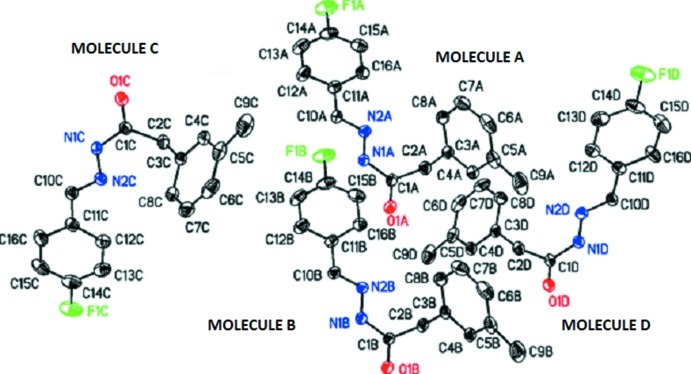
Molecular structure of the title compound with four molecules (A, B, C, D) in the asymmetric unit showing the atom labeling scheme and 30% probability displacement ellipsoids.

**Fig. 2.**
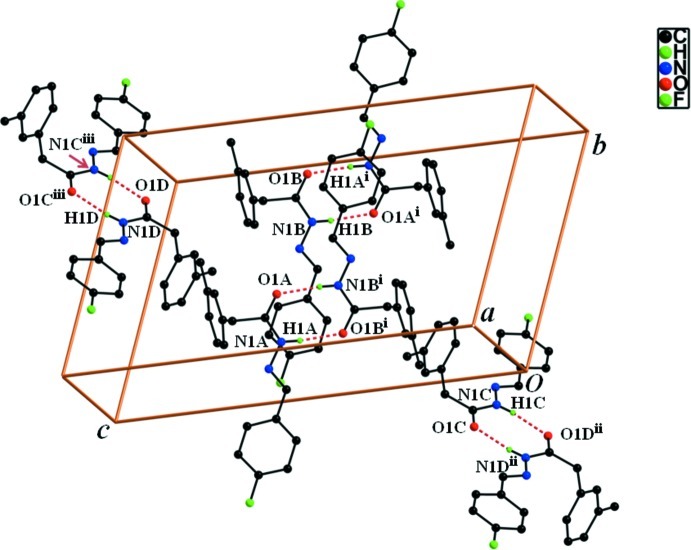
Packing diagram of the title compound viewed along the a axis. Dashed lines indicate N—H···O hydrogen bonds linkng the molecules into 1-D chains along [010] The remaining H atoms have been removed for clarity. [Symmetry code: (i) - x + 1, - y + 1, - z + 1; (ii) x - 1, y - 1, z - 1]

## supplementary crystallographic information

### Comment

Schiff bases are used as substrates in the preparation of number of industrial and biologically active compounds via ring closure, cycloaddition and replacement reactions. Schiff bases have also been employed as ligands for complexation of metal ions (Aydogan *et al.*, 2001). On the industrial scale, they have wide range of applications such as dyes and pigments (Taggi *et al.*, 2002). Compounds containing an azine functionality or a diimine linkage have been investigated in terms of their crystallography and coordination chemistry (Xu *et al.*, 1997; Kundu *et al.*, 2005). The crystal structures of some Schiff base hydrazines, viz., 4-fluorobenzaldehyde [(E)-4-fluorobenzylidene]hydrazone (Odabaşoğlu *et al.*, 2007), N'-[(E)-1-(4-bromophenyl)ethylidene]-2-(2-methyl-4-nitro-1H-imidazol-1-yl) acetohydrazide, (Fun *et al.*, 2012) N'-(4-Chlorobenzylidene)-2-[4-(methylsulfanyl)phenyl]acetohydrazide, N'-(4-fluorobenzylidene)-2-(4-fluorophenyl)acetohydrazide (Fun *et al.*, 2011*a*,*b*) and 2-(1H-1,2,3-benzotriazol-1-yl)-N'-(2-chlorobenzylidene)acetohydrazide (He & Shi, 2011) have been reported. In view of the importance of Schiff base hydrazines, the crystal structure of title compound is reported.

In the title compound, four molecules (A, B, C, D) crystallize in the asymmetric unit (Fig. 1). The dihedral angle between the benzene rings are twisted by 74.7 (8)° (A), 74.1 (1)° (B), 74.2 (7)° (C) and 74.3 (5)° (D), respectively. Bond lengths are in normal ranges (Allen *et al.*, 1987). In the crystal, N—H—O hydrogen bonds (Table 1) between hydrazide and aceto groups in nearby molecules forming an R 2,2(18) ring motif structure link the molecules into dimers which form columns along [010] (Fig. 2).

### Experimental

To a stirred solution of 2-m-tolylacetohydrazide (1g, 6.09 mmol) in ethanol (10 mL), 4-fluorobenzaldehyde (0.76 g, 6.09 mmol) was added and strirred at room temperature for 30 minutes (Fig. 3). Precipitated solid was filtered and dried. The single crystal was grown from toluene by the slow evaporation method and yield of the compound was 92%. (m.p.: 401–403 K).

### Refinement

All of the H atoms were placed in their calculated positions and then refined using the riding model with Atom—H lengths of 0.93Å (CH), 0.97Å (CH_2_), 0.96Å (CH_3_) or 0.86Å (NH). Isotropic displacement parameters for these atoms were set to 1.19-1.21 (CH, CH_2_), 1.49 (CH_3_) or 1.20 (NH) times *U*_eq_ of the parent atom.

### Crystal data

**Table:**

C_16_H_15_FN_2_O	*Z* = 8
*M**_r_* = 270.30	*F*(000) = 1136
Triclinic, *P*1	*D*_x_ = 1.279 Mg m^−^^3^
Hall symbol: -P 1	Cu *K*α radiation, λ = 1.54184 Å
*a* = 11.8535 (7) Å	Cell parameters from 4137 reflections
*b* = 12.3769 (9) Å	θ = 3.8–72.4°
*c* = 20.8721 (11) Å	µ = 0.74 mm^−^^1^
α = 98.549 (5)°	*T* = 173 K
β = 103.074 (5)°	Chunk, colorless
γ = 105.134 (6)°	0.22 × 0.16 × 0.08 mm
*V* = 2808.2 (3) Å^3^	

### Data collection

**Table:**

Agilent Xcalibur (Eos, Gemini) diffractometer	9250 independent reflections
Radiation source: Enhance (Cu) X-ray Source	3807 reflections with *I* > 2σ(*I*)
Graphite monochromator	*R*_int_ = 0.027
Detector resolution: 16.0416 pixels mm^-1^	θ_max_ = 63.7°, θ_min_ = 3.8°
ω scans	*h* = −12→13
Absorption correction: multi-scan (*CrysAlis PRO* and *CrysAlis RED*; Agilent, 2012)	*k* = −14→13
*T*_min_ = 0.780, *T*_max_ = 1.000	*l* = −16→24
16410 measured reflections	

### Refinement

**Table:**

Refinement on *F*^2^	Primary atom site location: structure-invariant direct methods
Least-squares matrix: full	Secondary atom site location: difference Fourier map
*R*[*F*^2^ > 2σ(*F*^2^)] = 0.051	Hydrogen site location: inferred from neighbouring sites
*wR*(*F*^2^) = 0.191	H-atom parameters constrained
*S* = 0.99	*w* = 1/[σ^2^(*F*_o_^2^) + (0.0836*P*)^2^] where *P* = (*F*_o_^2^ + 2*F*_c_^2^)/3
9250 reflections	(Δ/σ)_max_ < 0.001
725 parameters	Δρ_max_ = 0.19 e Å^−^^3^
0 restraints	Δρ_min_ = −0.22 e Å^−^^3^

### Special details

**Table:**

Geometry. All esds (except the esd in the dihedral angle between two l.s. planes) are estimated using the full covariance matrix. The cell esds are taken into account individually in the estimation of esds in distances, angles and torsion angles; correlations between esds in cell parameters are only used when they are defined by crystal symmetry. An approximate (isotropic) treatment of cell esds is used for estimating esds involving l.s. planes.
Refinement. Refinement of *F*^2^ against ALL reflections. The weighted *R*-factor *wR* and goodness of fit *S* are based on *F*^2^, conventional *R*-factors *R* are based on *F*, with *F* set to zero for negative *F*^2^. The threshold expression of *F*^2^ > σ(*F*^2^) is used only for calculating *R*-factors(gt) *etc*. and is not relevant to the choice of reflections for refinement. *R*-factors based on *F*^2^ are statistically about twice as large as those based on *F*, and *R*- factors based on ALL data will be even larger.

### Fractional atomic coordinates and isotropic or equivalent isotropic displacement parameters (Å^2^)

**Table:**

	*x*	*y*	*z*	*U*_iso_*/*U*_eq_	
F1A	0.1350 (2)	−0.4488 (2)	0.58889 (13)	0.0873 (9)	
O1A	0.6076 (2)	0.3277 (2)	0.57816 (11)	0.0437 (7)	
N1A	0.4866 (2)	0.1468 (2)	0.55590 (13)	0.0366 (7)	
H1A	0.4592	0.1490	0.5145	0.044*	
N2A	0.4417 (3)	0.0477 (2)	0.57697 (13)	0.0383 (8)	
C1A	0.5722 (3)	0.2402 (3)	0.59807 (17)	0.0366 (9)	
C2A	0.6254 (3)	0.2316 (3)	0.66978 (16)	0.0424 (10)	
H2AA	0.5718	0.1678	0.6809	0.051*	
H2AB	0.6343	0.3014	0.7012	0.051*	
C3A	0.7487 (4)	0.2136 (3)	0.67480 (16)	0.0380 (9)	
C4A	0.8535 (3)	0.3072 (3)	0.69544 (16)	0.0421 (9)	
H4A	0.8477	0.3799	0.7097	0.051*	
C5A	0.9663 (4)	0.2952 (4)	0.69533 (19)	0.0549 (12)	
C6A	0.9726 (4)	0.1853 (4)	0.67436 (19)	0.0642 (14)	
H6A	1.0473	0.1746	0.6739	0.077*	
C7A	0.8674 (4)	0.0909 (4)	0.65398 (19)	0.0579 (12)	
H7A	0.8728	0.0180	0.6400	0.069*	
C8A	0.7564 (4)	0.1044 (3)	0.65430 (16)	0.0473 (11)	
H8A	0.6869	0.0411	0.6409	0.057*	
C9A	1.0773 (4)	0.3989 (4)	0.7144 (2)	0.0813 (16)	
H9AA	1.0909	0.4229	0.6744	0.122*	
H9AB	1.1467	0.3798	0.7375	0.122*	
H9AC	1.0651	0.4600	0.7434	0.122*	
C10A	0.3539 (3)	−0.0306 (3)	0.53391 (17)	0.0393 (9)	
H10A	0.3245	−0.0180	0.4914	0.047*	
C11A	0.2985 (4)	−0.1393 (3)	0.54956 (18)	0.0435 (10)	
C12A	0.1988 (4)	−0.2200 (4)	0.5016 (2)	0.0602 (12)	
H12A	0.1694	−0.2034	0.4602	0.072*	
C13A	0.1427 (4)	−0.3244 (4)	0.5145 (2)	0.0693 (14)	
H13A	0.0763	−0.3780	0.4825	0.083*	
C14A	0.1884 (4)	−0.3459 (4)	0.5761 (2)	0.0607 (12)	
C15A	0.2864 (4)	−0.2689 (4)	0.6243 (2)	0.0583 (12)	
H15A	0.3154	−0.2865	0.6654	0.070*	
C16A	0.3415 (4)	−0.1650 (3)	0.61114 (19)	0.0476 (10)	
H16A	0.4077	−0.1120	0.6437	0.057*	
F1B	0.1356 (2)	0.0510 (2)	0.58903 (13)	0.0886 (9)	
O1B	0.6075 (2)	0.8278 (2)	0.57834 (11)	0.0440 (7)	
N1B	0.4862 (2)	0.6464 (2)	0.55557 (13)	0.0384 (8)	
H1B	0.4589	0.6486	0.5141	0.046*	
N2B	0.4414 (3)	0.5482 (2)	0.57698 (13)	0.0368 (7)	
C1B	0.5719 (3)	0.7395 (3)	0.59798 (17)	0.0358 (9)	
C2B	0.6264 (3)	0.7321 (3)	0.66962 (15)	0.0420 (10)	
H2BA	0.6361	0.8026	0.7007	0.050*	
H2BB	0.5729	0.6691	0.6814	0.050*	
C3B	0.7486 (3)	0.7131 (3)	0.67475 (15)	0.0368 (9)	
C4B	0.8532 (3)	0.8068 (3)	0.69576 (16)	0.0407 (9)	
H4B	0.8476	0.8794	0.7106	0.049*	
C5B	0.9661 (4)	0.7944 (4)	0.69511 (19)	0.0531 (12)	
C6B	0.9718 (4)	0.6843 (4)	0.6741 (2)	0.0642 (13)	
H6B	1.0465	0.6737	0.6736	0.077*	
C7B	0.8696 (4)	0.5918 (4)	0.65430 (19)	0.0608 (13)	
H7B	0.8753	0.5189	0.6404	0.073*	
C8B	0.7582 (4)	0.6052 (3)	0.65471 (16)	0.0481 (11)	
H8B	0.6891	0.5414	0.6415	0.058*	
C9B	1.0768 (4)	0.8985 (4)	0.7144 (2)	0.0846 (17)	
H9BA	1.0519	0.9664	0.7159	0.127*	
H9BB	1.1216	0.8942	0.6816	0.127*	
H9BC	1.1273	0.9011	0.7580	0.127*	
C10B	0.3550 (3)	0.4701 (3)	0.53307 (17)	0.0399 (9)	
H10B	0.3280	0.4825	0.4902	0.048*	
C11B	0.2973 (4)	0.3610 (3)	0.54858 (18)	0.0414 (9)	
C12B	0.1991 (4)	0.2800 (4)	0.5017 (2)	0.0608 (12)	
H12B	0.1693	0.2963	0.4602	0.073*	
C13B	0.1434 (4)	0.1756 (4)	0.5140 (2)	0.0683 (14)	
H13B	0.0774	0.1219	0.4816	0.082*	
C14B	0.1884 (4)	0.1537 (4)	0.5750 (2)	0.0612 (13)	
C15B	0.2858 (4)	0.2294 (4)	0.6240 (2)	0.0599 (12)	
H15B	0.3141	0.2114	0.6652	0.072*	
C16B	0.3411 (4)	0.3340 (3)	0.61045 (18)	0.0466 (10)	
H16B	0.4078	0.3866	0.6428	0.056*	
F1C	−0.3648 (3)	0.3109 (2)	0.08860 (14)	0.0866 (9)	
O1C	0.1075 (2)	−0.2351 (2)	0.07803 (11)	0.0449 (7)	
N1C	−0.0140 (2)	−0.1252 (2)	0.05571 (13)	0.0367 (7)	
H1C	−0.0414	−0.1617	0.0142	0.044*	
N2C	−0.0584 (3)	−0.0385 (2)	0.07720 (13)	0.0368 (7)	
C1C	0.0715 (3)	−0.1546 (3)	0.09808 (17)	0.0382 (9)	
C2C	0.1263 (3)	−0.0834 (3)	0.16952 (16)	0.0419 (9)	
H2CA	0.0730	−0.0407	0.1810	0.050*	
H2CB	0.1353	−0.1332	0.2008	0.050*	
C3C	0.2487 (3)	−0.0017 (3)	0.17474 (15)	0.0375 (9)	
C4C	0.3534 (3)	−0.0315 (3)	0.19560 (16)	0.0421 (9)	
H4C	0.3476	−0.0994	0.2105	0.051*	
C5C	0.4671 (4)	0.0361 (4)	0.19508 (19)	0.0542 (11)	
C6C	0.4714 (4)	0.1393 (4)	0.17407 (19)	0.0654 (14)	
H6C	0.5456	0.1878	0.1736	0.079*	
C7C	0.3665 (4)	0.1700 (4)	0.15395 (18)	0.0582 (13)	
H7C	0.3712	0.2386	0.1400	0.070*	
C8C	0.2551 (4)	0.0999 (3)	0.15443 (16)	0.0457 (10)	
H8C	0.1852	0.1212	0.1412	0.055*	
C9C	0.5790 (4)	−0.0030 (4)	0.2150 (2)	0.0817 (16)	
H9CA	0.5814	−0.0568	0.1776	0.123*	
H9CB	0.5754	−0.0389	0.2525	0.123*	
H9CC	0.6508	0.0622	0.2276	0.123*	
C10C	−0.1451 (3)	−0.0259 (3)	0.03379 (17)	0.0417 (9)	
H10C	−0.1735	−0.0742	−0.0089	0.050*	
C11C	−0.2012 (4)	0.0632 (3)	0.04950 (18)	0.0433 (10)	
C12C	−0.1582 (4)	0.1419 (3)	0.11061 (19)	0.0482 (10)	
H12C	−0.0912	0.1394	0.1430	0.058*	
C13C	−0.2144 (4)	0.2244 (3)	0.1238 (2)	0.0589 (12)	
H13C	−0.1861	0.2763	0.1653	0.071*	
C14C	−0.3114 (5)	0.2293 (4)	0.0759 (3)	0.0619 (13)	
C15C	−0.3561 (4)	0.1544 (4)	0.0149 (2)	0.0676 (14)	
H15C	−0.4221	0.1591	−0.0173	0.081*	
C16C	−0.3010 (4)	0.0709 (4)	0.00196 (19)	0.0606 (12)	
H16C	−0.3311	0.0187	−0.0394	0.073*	
F1D	1.3651 (3)	0.1892 (2)	0.91119 (14)	0.0887 (9)	
O1D	0.8924 (2)	0.7349 (2)	0.92168 (11)	0.0439 (7)	
N1D	1.0131 (3)	0.6259 (2)	0.94424 (13)	0.0386 (8)	
H1D	1.0400	0.6625	0.9857	0.046*	
N2D	1.0586 (3)	0.5392 (2)	0.92337 (13)	0.0381 (8)	
C1D	0.9281 (3)	0.6553 (3)	0.90193 (16)	0.0352 (8)	
C2D	0.8742 (3)	0.5849 (3)	0.83039 (15)	0.0416 (10)	
H2DA	0.9277	0.5426	0.8188	0.050*	
H2DB	0.8648	0.6350	0.7993	0.050*	
C3D	0.7511 (3)	0.5021 (3)	0.82527 (15)	0.0372 (9)	
C4D	0.6466 (3)	0.5325 (3)	0.80431 (16)	0.0413 (9)	
H4D	0.6522	0.6006	0.7896	0.050*	
C5D	0.5344 (4)	0.4645 (4)	0.80461 (19)	0.0525 (11)	
C6D	0.5272 (4)	0.3623 (4)	0.8256 (2)	0.0642 (13)	
H6D	0.4521	0.3151	0.8258	0.077*	
C7D	0.6295 (4)	0.3298 (3)	0.84599 (19)	0.0604 (13)	
H7D	0.6233	0.2612	0.8602	0.072*	
C8D	0.7410 (4)	0.3984 (3)	0.84551 (16)	0.0485 (11)	
H8D	0.8099	0.3755	0.8587	0.058*	
C9D	0.4222 (4)	0.5016 (4)	0.7854 (2)	0.0811 (16)	
H9DA	0.4022	0.5297	0.8255	0.122*	
H9DB	0.3555	0.4374	0.7575	0.122*	
H9DC	0.4373	0.5615	0.7611	0.122*	
C10D	1.1461 (3)	0.5258 (3)	0.96689 (16)	0.0397 (9)	
H10D	1.1744	0.5736	1.0097	0.048*	
C11D	1.2027 (3)	0.4376 (3)	0.95104 (18)	0.0419 (9)	
C12D	1.1598 (4)	0.3588 (3)	0.88932 (19)	0.0490 (10)	
H12D	1.0936	0.3623	0.8567	0.059*	
C13D	1.2147 (4)	0.2754 (3)	0.8760 (2)	0.0609 (12)	
H13D	1.1864	0.2226	0.8349	0.073*	
C14D	1.3122 (4)	0.2728 (4)	0.9252 (2)	0.0602 (13)	
C15D	1.3568 (4)	0.3485 (4)	0.9863 (2)	0.0713 (14)	
H15D	1.4236	0.3452	1.0185	0.086*	
C16D	1.3001 (4)	0.4297 (4)	0.99869 (19)	0.0581 (12)	
H16D	1.3282	0.4808	1.0404	0.070*	

### Atomic displacement parameters (Å^2^)

**Table:**

	*U*^11^	*U*^22^	*U*^33^	*U*^12^	*U*^13^	*U*^23^
F1A	0.107 (2)	0.0493 (17)	0.119 (2)	0.0109 (16)	0.0676 (19)	0.0286 (16)
O1A	0.0440 (16)	0.0344 (15)	0.0420 (14)	0.0024 (12)	0.0028 (12)	0.0073 (12)
N1A	0.0375 (19)	0.0345 (18)	0.0312 (16)	0.0073 (14)	0.0019 (14)	0.0064 (14)
N2A	0.0387 (19)	0.0354 (19)	0.0388 (17)	0.0079 (15)	0.0107 (15)	0.0078 (15)
C1A	0.034 (2)	0.038 (2)	0.034 (2)	0.0094 (17)	0.0054 (17)	0.0028 (17)
C2A	0.044 (2)	0.044 (2)	0.035 (2)	0.0111 (19)	0.0073 (19)	0.0077 (17)
C3A	0.047 (2)	0.037 (2)	0.0264 (19)	0.0099 (18)	0.0050 (18)	0.0084 (17)
C4A	0.046 (3)	0.042 (2)	0.035 (2)	0.0125 (19)	0.0045 (19)	0.0093 (17)
C5A	0.045 (3)	0.080 (4)	0.045 (2)	0.022 (3)	0.011 (2)	0.026 (2)
C6A	0.062 (3)	0.108 (4)	0.051 (3)	0.055 (3)	0.026 (2)	0.037 (3)
C7A	0.081 (4)	0.060 (3)	0.046 (2)	0.040 (3)	0.018 (3)	0.018 (2)
C8A	0.063 (3)	0.045 (3)	0.031 (2)	0.017 (2)	0.007 (2)	0.0087 (18)
C9A	0.045 (3)	0.116 (5)	0.079 (3)	0.009 (3)	0.010 (3)	0.045 (3)
C10A	0.037 (2)	0.042 (2)	0.035 (2)	0.0091 (18)	0.0066 (17)	0.0053 (17)
C11A	0.049 (3)	0.041 (2)	0.042 (2)	0.0127 (19)	0.020 (2)	0.0060 (19)
C12A	0.064 (3)	0.052 (3)	0.046 (2)	−0.007 (2)	0.012 (2)	0.004 (2)
C13A	0.077 (4)	0.046 (3)	0.071 (3)	−0.004 (2)	0.027 (3)	0.001 (3)
C14A	0.073 (4)	0.038 (3)	0.084 (3)	0.014 (2)	0.048 (3)	0.019 (2)
C15A	0.068 (3)	0.055 (3)	0.068 (3)	0.027 (2)	0.032 (3)	0.026 (2)
C16A	0.050 (3)	0.045 (3)	0.051 (2)	0.014 (2)	0.018 (2)	0.016 (2)
F1B	0.112 (2)	0.0460 (16)	0.124 (2)	0.0121 (16)	0.072 (2)	0.0309 (16)
O1B	0.0454 (16)	0.0391 (16)	0.0371 (14)	0.0038 (12)	0.0020 (12)	0.0079 (12)
N1B	0.0401 (19)	0.0368 (19)	0.0320 (16)	0.0053 (15)	0.0055 (15)	0.0070 (14)
N2B	0.043 (2)	0.0325 (18)	0.0324 (16)	0.0103 (15)	0.0078 (15)	0.0068 (14)
C1B	0.038 (2)	0.033 (2)	0.0335 (19)	0.0088 (17)	0.0079 (17)	0.0037 (16)
C2B	0.045 (2)	0.043 (2)	0.0285 (19)	0.0038 (19)	0.0066 (18)	0.0022 (16)
C3B	0.042 (2)	0.044 (2)	0.0217 (18)	0.0132 (19)	0.0041 (17)	0.0077 (16)
C4B	0.042 (2)	0.046 (2)	0.0298 (19)	0.0107 (19)	0.0042 (18)	0.0088 (17)
C5B	0.042 (3)	0.076 (3)	0.041 (2)	0.015 (2)	0.006 (2)	0.025 (2)
C6B	0.059 (3)	0.098 (4)	0.060 (3)	0.045 (3)	0.027 (3)	0.036 (3)
C7B	0.090 (4)	0.067 (3)	0.049 (3)	0.048 (3)	0.030 (3)	0.023 (2)
C8B	0.065 (3)	0.045 (3)	0.034 (2)	0.017 (2)	0.011 (2)	0.0106 (19)
C9B	0.042 (3)	0.110 (4)	0.089 (4)	0.001 (3)	0.003 (3)	0.048 (3)
C10B	0.048 (3)	0.036 (2)	0.033 (2)	0.0095 (19)	0.0121 (18)	0.0040 (17)
C11B	0.046 (2)	0.036 (2)	0.041 (2)	0.0086 (18)	0.0158 (19)	0.0031 (18)
C12B	0.072 (3)	0.047 (3)	0.051 (3)	−0.001 (2)	0.020 (2)	0.007 (2)
C13B	0.073 (4)	0.049 (3)	0.064 (3)	−0.012 (2)	0.026 (3)	−0.002 (2)
C14B	0.074 (4)	0.037 (3)	0.083 (3)	0.008 (2)	0.051 (3)	0.013 (2)
C15B	0.070 (3)	0.059 (3)	0.071 (3)	0.029 (3)	0.039 (3)	0.032 (3)
C16B	0.050 (3)	0.045 (3)	0.049 (2)	0.018 (2)	0.019 (2)	0.0085 (19)
F1C	0.108 (2)	0.0767 (19)	0.122 (2)	0.0641 (18)	0.0694 (19)	0.0417 (17)
O1C	0.0459 (17)	0.0461 (16)	0.0402 (14)	0.0205 (13)	0.0012 (13)	0.0066 (12)
N1C	0.0380 (19)	0.0382 (18)	0.0299 (16)	0.0136 (15)	0.0020 (14)	0.0037 (13)
N2C	0.0381 (19)	0.0402 (19)	0.0354 (16)	0.0165 (15)	0.0106 (15)	0.0103 (14)
C1C	0.039 (2)	0.038 (2)	0.037 (2)	0.0118 (18)	0.0080 (18)	0.0122 (17)
C2C	0.040 (2)	0.049 (2)	0.033 (2)	0.0143 (19)	0.0048 (18)	0.0060 (18)
C3C	0.047 (3)	0.040 (2)	0.0247 (18)	0.0169 (19)	0.0067 (17)	0.0036 (16)
C4C	0.045 (3)	0.042 (2)	0.034 (2)	0.0126 (19)	0.0051 (18)	0.0039 (17)
C5C	0.042 (3)	0.066 (3)	0.043 (2)	0.008 (2)	0.011 (2)	−0.006 (2)
C6C	0.070 (3)	0.057 (3)	0.050 (3)	−0.015 (3)	0.028 (2)	−0.004 (2)
C7C	0.082 (4)	0.048 (3)	0.041 (2)	0.017 (3)	0.017 (2)	0.006 (2)
C8C	0.058 (3)	0.043 (3)	0.032 (2)	0.016 (2)	0.008 (2)	0.0031 (18)
C9C	0.040 (3)	0.103 (4)	0.082 (3)	0.020 (3)	0.005 (3)	−0.019 (3)
C10C	0.047 (3)	0.047 (2)	0.035 (2)	0.019 (2)	0.0127 (19)	0.0087 (18)
C11C	0.050 (3)	0.051 (3)	0.039 (2)	0.022 (2)	0.019 (2)	0.0179 (19)
C12C	0.055 (3)	0.044 (3)	0.052 (2)	0.018 (2)	0.024 (2)	0.0136 (19)
C13C	0.071 (3)	0.049 (3)	0.067 (3)	0.021 (2)	0.036 (3)	0.010 (2)
C14C	0.076 (4)	0.057 (3)	0.085 (3)	0.041 (3)	0.052 (3)	0.031 (3)
C15C	0.069 (3)	0.090 (4)	0.076 (3)	0.056 (3)	0.032 (3)	0.042 (3)
C16C	0.070 (3)	0.074 (3)	0.045 (2)	0.038 (3)	0.011 (2)	0.014 (2)
F1D	0.110 (2)	0.083 (2)	0.121 (2)	0.0664 (18)	0.0681 (19)	0.0434 (17)
O1D	0.0444 (17)	0.0448 (16)	0.0394 (14)	0.0202 (13)	0.0018 (12)	0.0039 (12)
N1D	0.041 (2)	0.0401 (19)	0.0324 (16)	0.0161 (15)	0.0046 (15)	0.0039 (14)
N2D	0.041 (2)	0.0378 (19)	0.0346 (16)	0.0127 (15)	0.0084 (15)	0.0068 (14)
C1D	0.031 (2)	0.041 (2)	0.033 (2)	0.0126 (17)	0.0052 (17)	0.0082 (16)
C2D	0.043 (3)	0.051 (3)	0.0312 (19)	0.017 (2)	0.0074 (18)	0.0092 (18)
C3D	0.041 (2)	0.042 (2)	0.0225 (17)	0.0107 (18)	0.0037 (17)	0.0008 (16)
C4D	0.044 (2)	0.045 (2)	0.0310 (19)	0.0137 (19)	0.0050 (18)	0.0040 (17)
C5D	0.042 (3)	0.058 (3)	0.043 (2)	0.007 (2)	0.006 (2)	−0.010 (2)
C6D	0.055 (3)	0.061 (3)	0.058 (3)	−0.006 (2)	0.023 (2)	−0.010 (2)
C7D	0.086 (4)	0.036 (3)	0.051 (3)	0.000 (2)	0.027 (3)	0.006 (2)
C8D	0.073 (3)	0.045 (3)	0.030 (2)	0.027 (2)	0.010 (2)	0.0053 (18)
C9D	0.045 (3)	0.101 (4)	0.078 (3)	0.018 (3)	0.007 (3)	−0.015 (3)
C10D	0.041 (2)	0.044 (2)	0.033 (2)	0.0152 (18)	0.0046 (17)	0.0099 (17)
C11D	0.044 (3)	0.044 (2)	0.046 (2)	0.0172 (19)	0.018 (2)	0.0174 (19)
C12D	0.051 (3)	0.050 (3)	0.052 (2)	0.019 (2)	0.017 (2)	0.017 (2)
C13D	0.072 (3)	0.050 (3)	0.070 (3)	0.021 (2)	0.036 (3)	0.013 (2)
C14D	0.067 (3)	0.064 (3)	0.085 (3)	0.043 (3)	0.046 (3)	0.041 (3)
C15D	0.076 (4)	0.100 (4)	0.057 (3)	0.053 (3)	0.018 (3)	0.027 (3)
C16D	0.063 (3)	0.078 (3)	0.049 (2)	0.048 (3)	0.015 (2)	0.017 (2)

### Geometric parameters (Å, º)

**Table:**

F1A—C14A	1.361 (4)	F1C—C14C	1.350 (4)
O1A—C1A	1.223 (4)	O1C—C1C	1.236 (4)
N1A—C1A	1.350 (4)	N1C—C1C	1.351 (4)
N1A—N2A	1.380 (3)	N1C—N2C	1.374 (3)
N1A—H1A	0.8600	N1C—H1C	0.8600
N2A—C10A	1.273 (4)	N2C—C10C	1.267 (4)
C1A—C2A	1.518 (4)	C1C—C2C	1.515 (4)
C2A—C3A	1.519 (4)	C2C—C3C	1.507 (5)
C2A—H2AA	0.9700	C2C—H2CA	0.9700
C2A—H2AB	0.9700	C2C—H2CB	0.9700
C3A—C8A	1.388 (5)	C3C—C8C	1.374 (4)
C3A—C4A	1.389 (5)	C3C—C4C	1.382 (4)
C4A—C5A	1.383 (5)	C4C—C5C	1.392 (5)
C4A—H4A	0.9300	C4C—H4C	0.9300
C5A—C6A	1.393 (6)	C5C—C6C	1.403 (5)
C5A—C9A	1.505 (5)	C5C—C9C	1.520 (5)
C6A—C7A	1.397 (6)	C6C—C7C	1.387 (5)
C6A—H6A	0.9300	C6C—H6C	0.9300
C7A—C8A	1.370 (5)	C7C—C8C	1.381 (5)
C7A—H7A	0.9300	C7C—H7C	0.9300
C8A—H8A	0.9300	C8C—H8C	0.9300
C9A—H9AA	0.9600	C9C—H9CA	0.9600
C9A—H9AB	0.9600	C9C—H9CB	0.9600
C9A—H9AC	0.9600	C9C—H9CC	0.9600
C10A—C11A	1.454 (5)	C10C—C11C	1.463 (5)
C10A—H10A	0.9300	C10C—H10C	0.9300
C11A—C16A	1.386 (4)	C11C—C12C	1.383 (5)
C11A—C12A	1.395 (5)	C11C—C16C	1.396 (5)
C12A—C13A	1.386 (5)	C12C—C13C	1.384 (5)
C12A—H12A	0.9300	C12C—H12C	0.9300
C13A—C14A	1.370 (5)	C13C—C14C	1.364 (5)
C13A—H13A	0.9300	C13C—H13C	0.9300
C14A—C15A	1.370 (6)	C14C—C15C	1.360 (6)
C15A—C16A	1.380 (5)	C15C—C16C	1.385 (5)
C15A—H15A	0.9300	C15C—H15C	0.9300
C16A—H16A	0.9300	C16C—H16C	0.9300
F1B—C14B	1.368 (4)	F1D—C14D	1.371 (4)
O1B—C1B	1.229 (4)	O1D—C1D	1.222 (4)
N1B—C1B	1.349 (4)	N1D—C1D	1.346 (4)
N1B—N2B	1.376 (3)	N1D—N2D	1.379 (3)
N1B—H1B	0.8600	N1D—H1D	0.8600
N2B—C10B	1.272 (4)	N2D—C10D	1.279 (4)
C1B—C2B	1.515 (4)	C1D—C2D	1.513 (4)
C2B—C3B	1.509 (4)	C2D—C3D	1.519 (5)
C2B—H2BA	0.9700	C2D—H2DA	0.9700
C2B—H2BB	0.9700	C2D—H2DB	0.9700
C3B—C8B	1.381 (5)	C3D—C4D	1.383 (4)
C3B—C4B	1.388 (5)	C3D—C8D	1.395 (4)
C4B—C5B	1.389 (5)	C4D—C5D	1.378 (5)
C4B—H4B	0.9300	C4D—H4D	0.9300
C5B—C6B	1.392 (6)	C5D—C6D	1.387 (5)
C5B—C9B	1.505 (5)	C5D—C9D	1.508 (5)
C6B—C7B	1.363 (6)	C6D—C7D	1.373 (5)
C6B—H6B	0.9300	C6D—H6D	0.9300
C7B—C8B	1.375 (5)	C7D—C8D	1.375 (5)
C7B—H7B	0.9300	C7D—H7D	0.9300
C8B—H8B	0.9300	C8D—H8D	0.9300
C9B—H9BA	0.9600	C9D—H9DA	0.9600
C9B—H9BB	0.9600	C9D—H9DB	0.9600
C9B—H9BC	0.9600	C9D—H9DC	0.9600
C10B—C11B	1.463 (4)	C10D—C11D	1.459 (4)
C10B—H10B	0.9300	C10D—H10D	0.9300
C11B—C12B	1.380 (5)	C11D—C16D	1.378 (5)
C11B—C16B	1.402 (4)	C11D—C12D	1.393 (5)
C12B—C13B	1.379 (5)	C12D—C13D	1.383 (5)
C12B—H12B	0.9300	C12D—H12D	0.9300
C13B—C14B	1.360 (5)	C13D—C14D	1.371 (5)
C13B—H13B	0.9300	C13D—H13D	0.9300
C14B—C15B	1.369 (6)	C14D—C15D	1.366 (6)
C15B—C16B	1.391 (5)	C15D—C16D	1.371 (5)
C15B—H15B	0.9300	C15D—H15D	0.9300
C16B—H16B	0.9300	C16D—H16D	0.9300
			
C1A—N1A—N2A	122.3 (3)	C1C—N1C—N2C	121.7 (3)
C1A—N1A—H1A	118.9	C1C—N1C—H1C	119.1
N2A—N1A—H1A	118.9	N2C—N1C—H1C	119.1
C10A—N2A—N1A	116.1 (3)	C10C—N2C—N1C	115.2 (3)
O1A—C1A—N1A	120.6 (3)	O1C—C1C—N1C	120.5 (3)
O1A—C1A—C2A	121.5 (3)	O1C—C1C—C2C	120.9 (3)
N1A—C1A—C2A	117.9 (3)	N1C—C1C—C2C	118.5 (3)
C1A—C2A—C3A	108.2 (3)	C3C—C2C—C1C	109.1 (3)
C1A—C2A—H2AA	110.1	C3C—C2C—H2CA	109.9
C3A—C2A—H2AA	110.1	C1C—C2C—H2CA	109.9
C1A—C2A—H2AB	110.1	C3C—C2C—H2CB	109.9
C3A—C2A—H2AB	110.1	C1C—C2C—H2CB	109.9
H2AA—C2A—H2AB	108.4	H2CA—C2C—H2CB	108.3
C8A—C3A—C4A	119.7 (4)	C8C—C3C—C4C	119.9 (4)
C8A—C3A—C2A	120.1 (4)	C8C—C3C—C2C	119.5 (3)
C4A—C3A—C2A	120.1 (3)	C4C—C3C—C2C	120.4 (3)
C5A—C4A—C3A	121.8 (4)	C3C—C4C—C5C	122.5 (4)
C5A—C4A—H4A	119.1	C3C—C4C—H4C	118.8
C3A—C4A—H4A	119.1	C5C—C4C—H4C	118.8
C4A—C5A—C6A	118.0 (4)	C4C—C5C—C6C	116.5 (4)
C4A—C5A—C9A	120.6 (4)	C4C—C5C—C9C	120.8 (4)
C6A—C5A—C9A	121.4 (4)	C6C—C5C—C9C	122.7 (4)
C5A—C6A—C7A	120.4 (4)	C7C—C6C—C5C	121.0 (4)
C5A—C6A—H6A	119.8	C7C—C6C—H6C	119.5
C7A—C6A—H6A	119.8	C5C—C6C—H6C	119.5
C8A—C7A—C6A	120.9 (4)	C8C—C7C—C6C	120.8 (4)
C8A—C7A—H7A	119.6	C8C—C7C—H7C	119.6
C6A—C7A—H7A	119.6	C6C—C7C—H7C	119.6
C7A—C8A—C3A	119.4 (4)	C3C—C8C—C7C	119.3 (4)
C7A—C8A—H8A	120.3	C3C—C8C—H8C	120.4
C3A—C8A—H8A	120.3	C7C—C8C—H8C	120.4
C5A—C9A—H9AA	109.5	C5C—C9C—H9CA	109.5
C5A—C9A—H9AB	109.5	C5C—C9C—H9CB	109.5
H9AA—C9A—H9AB	109.5	H9CA—C9C—H9CB	109.5
C5A—C9A—H9AC	109.5	C5C—C9C—H9CC	109.5
H9AA—C9A—H9AC	109.5	H9CA—C9C—H9CC	109.5
H9AB—C9A—H9AC	109.5	H9CB—C9C—H9CC	109.5
N2A—C10A—C11A	121.7 (3)	N2C—C10C—C11C	121.3 (3)
N2A—C10A—H10A	119.1	N2C—C10C—H10C	119.4
C11A—C10A—H10A	119.1	C11C—C10C—H10C	119.4
C16A—C11A—C12A	118.9 (4)	C12C—C11C—C16C	118.0 (4)
C16A—C11A—C10A	121.8 (4)	C12C—C11C—C10C	122.1 (3)
C12A—C11A—C10A	119.3 (3)	C16C—C11C—C10C	120.0 (4)
C13A—C12A—C11A	121.2 (4)	C11C—C12C—C13C	120.4 (4)
C13A—C12A—H12A	119.4	C11C—C12C—H12C	119.8
C11A—C12A—H12A	119.4	C13C—C12C—H12C	119.8
C14A—C13A—C12A	117.8 (4)	C14C—C13C—C12C	119.8 (4)
C14A—C13A—H13A	121.1	C14C—C13C—H13C	120.1
C12A—C13A—H13A	121.1	C12C—C13C—H13C	120.1
F1A—C14A—C15A	119.2 (4)	F1C—C14C—C15C	118.5 (4)
F1A—C14A—C13A	118.2 (4)	F1C—C14C—C13C	119.7 (5)
C15A—C14A—C13A	122.6 (4)	C15C—C14C—C13C	121.9 (4)
C14A—C15A—C16A	119.3 (4)	C14C—C15C—C16C	118.3 (4)
C14A—C15A—H15A	120.3	C14C—C15C—H15C	120.8
C16A—C15A—H15A	120.3	C16C—C15C—H15C	120.8
C15A—C16A—C11A	120.2 (4)	C15C—C16C—C11C	121.6 (4)
C15A—C16A—H16A	119.9	C15C—C16C—H16C	119.2
C11A—C16A—H16A	119.9	C11C—C16C—H16C	119.2
C1B—N1B—N2B	121.7 (3)	C1D—N1D—N2D	122.3 (3)
C1B—N1B—H1B	119.1	C1D—N1D—H1D	118.8
N2B—N1B—H1B	119.1	N2D—N1D—H1D	118.8
C10B—N2B—N1B	115.1 (3)	C10D—N2D—N1D	116.3 (3)
O1B—C1B—N1B	120.6 (3)	O1D—C1D—N1D	120.5 (3)
O1B—C1B—C2B	120.5 (3)	O1D—C1D—C2D	120.8 (3)
N1B—C1B—C2B	118.9 (3)	N1D—C1D—C2D	118.6 (3)
C3B—C2B—C1B	108.9 (3)	C1D—C2D—C3D	108.6 (3)
C3B—C2B—H2BA	109.9	C1D—C2D—H2DA	110.0
C1B—C2B—H2BA	109.9	C3D—C2D—H2DA	110.0
C3B—C2B—H2BB	109.9	C1D—C2D—H2DB	110.0
C1B—C2B—H2BB	109.9	C3D—C2D—H2DB	110.0
H2BA—C2B—H2BB	108.3	H2DA—C2D—H2DB	108.4
C8B—C3B—C4B	119.0 (4)	C4D—C3D—C8D	118.6 (4)
C8B—C3B—C2B	121.4 (4)	C4D—C3D—C2D	119.8 (3)
C4B—C3B—C2B	119.5 (3)	C8D—C3D—C2D	121.4 (3)
C3B—C4B—C5B	121.4 (4)	C5D—C4D—C3D	121.7 (4)
C3B—C4B—H4B	119.3	C5D—C4D—H4D	119.2
C5B—C4B—H4B	119.3	C3D—C4D—H4D	119.2
C4B—C5B—C6B	117.9 (4)	C4D—C5D—C6D	118.5 (4)
C4B—C5B—C9B	120.1 (4)	C4D—C5D—C9D	121.2 (4)
C6B—C5B—C9B	122.0 (4)	C6D—C5D—C9D	120.3 (4)
C7B—C6B—C5B	121.0 (4)	C7D—C6D—C5D	120.9 (4)
C7B—C6B—H6B	119.5	C7D—C6D—H6D	119.6
C5B—C6B—H6B	119.5	C5D—C6D—H6D	119.6
C6B—C7B—C8B	120.6 (4)	C6D—C7D—C8D	120.1 (4)
C6B—C7B—H7B	119.7	C6D—C7D—H7D	119.9
C8B—C7B—H7B	119.7	C8D—C7D—H7D	119.9
C7B—C8B—C3B	120.1 (4)	C7D—C8D—C3D	120.2 (4)
C7B—C8B—H8B	119.9	C7D—C8D—H8D	119.9
C3B—C8B—H8B	119.9	C3D—C8D—H8D	119.9
C5B—C9B—H9BA	109.5	C5D—C9D—H9DA	109.5
C5B—C9B—H9BB	109.5	C5D—C9D—H9DB	109.5
H9BA—C9B—H9BB	109.5	H9DA—C9D—H9DB	109.5
C5B—C9B—H9BC	109.5	C5D—C9D—H9DC	109.5
H9BA—C9B—H9BC	109.5	H9DA—C9D—H9DC	109.5
H9BB—C9B—H9BC	109.5	H9DB—C9D—H9DC	109.5
N2B—C10B—C11B	121.4 (3)	N2D—C10D—C11D	121.7 (3)
N2B—C10B—H10B	119.3	N2D—C10D—H10D	119.2
C11B—C10B—H10B	119.3	C11D—C10D—H10D	119.2
C12B—C11B—C16B	117.7 (4)	C16D—C11D—C12D	118.5 (4)
C12B—C11B—C10B	120.7 (3)	C16D—C11D—C10D	119.5 (4)
C16B—C11B—C10B	121.6 (4)	C12D—C11D—C10D	122.0 (3)
C13B—C12B—C11B	122.4 (4)	C13D—C12D—C11D	120.6 (4)
C13B—C12B—H12B	118.8	C13D—C12D—H12D	119.7
C11B—C12B—H12B	118.8	C11D—C12D—H12D	119.7
C14B—C13B—C12B	117.7 (4)	C14D—C13D—C12D	118.2 (4)
C14B—C13B—H13B	121.2	C14D—C13D—H13D	120.9
C12B—C13B—H13B	121.2	C12D—C13D—H13D	120.9
C13B—C14B—F1B	119.4 (4)	C15D—C14D—F1D	119.8 (4)
C13B—C14B—C15B	123.4 (4)	C15D—C14D—C13D	122.9 (4)
F1B—C14B—C15B	117.2 (4)	F1D—C14D—C13D	117.3 (4)
C14B—C15B—C16B	118.0 (4)	C14D—C15D—C16D	117.9 (4)
C14B—C15B—H15B	121.0	C14D—C15D—H15D	121.0
C16B—C15B—H15B	121.0	C16D—C15D—H15D	121.0
C15B—C16B—C11B	120.8 (4)	C15D—C16D—C11D	121.9 (4)
C15B—C16B—H16B	119.6	C15D—C16D—H16D	119.1
C11B—C16B—H16B	119.6	C11D—C16D—H16D	119.1
			
C1A—N1A—N2A—C10A	174.1 (3)	C1C—N1C—N2C—C10C	−174.6 (3)
N2A—N1A—C1A—O1A	−178.0 (3)	N2C—N1C—C1C—O1C	177.9 (3)
N2A—N1A—C1A—C2A	4.0 (5)	N2C—N1C—C1C—C2C	−4.9 (5)
O1A—C1A—C2A—C3A	−76.9 (4)	O1C—C1C—C2C—C3C	76.7 (4)
N1A—C1A—C2A—C3A	101.1 (4)	N1C—C1C—C2C—C3C	−100.6 (4)
C1A—C2A—C3A—C8A	−82.7 (4)	C1C—C2C—C3C—C8C	82.4 (4)
C1A—C2A—C3A—C4A	92.8 (4)	C1C—C2C—C3C—C4C	−93.4 (4)
C8A—C3A—C4A—C5A	0.9 (5)	C8C—C3C—C4C—C5C	−2.0 (6)
C2A—C3A—C4A—C5A	−174.6 (3)	C2C—C3C—C4C—C5C	173.7 (3)
C3A—C4A—C5A—C6A	−0.6 (6)	C3C—C4C—C5C—C6C	1.9 (6)
C3A—C4A—C5A—C9A	176.7 (3)	C3C—C4C—C5C—C9C	−176.8 (3)
C4A—C5A—C6A—C7A	0.2 (6)	C4C—C5C—C6C—C7C	−1.1 (6)
C9A—C5A—C6A—C7A	−177.1 (4)	C9C—C5C—C6C—C7C	177.7 (3)
C5A—C6A—C7A—C8A	−0.1 (6)	C5C—C6C—C7C—C8C	0.3 (6)
C6A—C7A—C8A—C3A	0.4 (6)	C4C—C3C—C8C—C7C	1.2 (5)
C4A—C3A—C8A—C7A	−0.8 (5)	C2C—C3C—C8C—C7C	−174.6 (3)
C2A—C3A—C8A—C7A	174.7 (3)	C6C—C7C—C8C—C3C	−0.4 (6)
N1A—N2A—C10A—C11A	179.8 (3)	N1C—N2C—C10C—C11C	−179.9 (3)
N2A—C10A—C11A—C16A	−2.7 (6)	N2C—C10C—C11C—C12C	3.9 (6)
N2A—C10A—C11A—C12A	177.0 (4)	N2C—C10C—C11C—C16C	−176.4 (3)
C16A—C11A—C12A—C13A	0.0 (7)	C16C—C11C—C12C—C13C	0.8 (6)
C10A—C11A—C12A—C13A	−179.6 (4)	C10C—C11C—C12C—C13C	−179.5 (3)
C11A—C12A—C13A—C14A	0.0 (7)	C11C—C12C—C13C—C14C	−1.1 (6)
C12A—C13A—C14A—F1A	−179.1 (4)	C12C—C13C—C14C—F1C	−179.1 (3)
C12A—C13A—C14A—C15A	−0.3 (7)	C12C—C13C—C14C—C15C	0.6 (7)
F1A—C14A—C15A—C16A	179.3 (3)	F1C—C14C—C15C—C16C	179.9 (4)
C13A—C14A—C15A—C16A	0.5 (7)	C13C—C14C—C15C—C16C	0.2 (7)
C14A—C15A—C16A—C11A	−0.4 (6)	C14C—C15C—C16C—C11C	−0.5 (7)
C12A—C11A—C16A—C15A	0.2 (6)	C12C—C11C—C16C—C15C	0.0 (6)
C10A—C11A—C16A—C15A	179.8 (4)	C10C—C11C—C16C—C15C	−179.7 (4)
C1B—N1B—N2B—C10B	175.3 (3)	C1D—N1D—N2D—C10D	174.4 (3)
N2B—N1B—C1B—O1B	−177.4 (3)	N2D—N1D—C1D—O1D	−177.8 (3)
N2B—N1B—C1B—C2B	4.6 (5)	N2D—N1D—C1D—C2D	4.6 (5)
O1B—C1B—C2B—C3B	−77.7 (4)	O1D—C1D—C2D—C3D	−77.3 (4)
N1B—C1B—C2B—C3B	100.3 (4)	N1D—C1D—C2D—C3D	100.3 (4)
C1B—C2B—C3B—C8B	−81.8 (4)	C1D—C2D—C3D—C4D	93.7 (4)
C1B—C2B—C3B—C4B	93.8 (4)	C1D—C2D—C3D—C8D	−82.0 (4)
C8B—C3B—C4B—C5B	1.9 (5)	C8D—C3D—C4D—C5D	1.7 (5)
C2B—C3B—C4B—C5B	−173.8 (3)	C2D—C3D—C4D—C5D	−174.2 (3)
C3B—C4B—C5B—C6B	−1.5 (5)	C3D—C4D—C5D—C6D	−1.1 (6)
C3B—C4B—C5B—C9B	176.5 (3)	C3D—C4D—C5D—C9D	176.5 (3)
C4B—C5B—C6B—C7B	0.6 (6)	C4D—C5D—C6D—C7D	0.4 (6)
C9B—C5B—C6B—C7B	−177.3 (4)	C9D—C5D—C6D—C7D	−177.1 (4)
C5B—C6B—C7B—C8B	−0.1 (6)	C5D—C6D—C7D—C8D	−0.4 (6)
C6B—C7B—C8B—C3B	0.6 (6)	C6D—C7D—C8D—C3D	1.0 (6)
C4B—C3B—C8B—C7B	−1.4 (5)	C4D—C3D—C8D—C7D	−1.6 (5)
C2B—C3B—C8B—C7B	174.2 (3)	C2D—C3D—C8D—C7D	174.2 (3)
N1B—N2B—C10B—C11B	−179.5 (3)	N1D—N2D—C10D—C11D	−179.6 (3)
N2B—C10B—C11B—C12B	176.1 (4)	N2D—C10D—C11D—C16D	177.3 (3)
N2B—C10B—C11B—C16B	−5.4 (6)	N2D—C10D—C11D—C12D	−4.1 (6)
C16B—C11B—C12B—C13B	0.5 (7)	C16D—C11D—C12D—C13D	−0.7 (6)
C10B—C11B—C12B—C13B	179.0 (4)	C10D—C11D—C12D—C13D	−179.4 (3)
C11B—C12B—C13B—C14B	0.1 (7)	C11D—C12D—C13D—C14D	0.0 (6)
C12B—C13B—C14B—F1B	−180.0 (4)	C12D—C13D—C14D—C15D	0.1 (7)
C12B—C13B—C14B—C15B	−0.5 (7)	C12D—C13D—C14D—F1D	179.7 (3)
C13B—C14B—C15B—C16B	0.1 (7)	F1D—C14D—C15D—C16D	−179.0 (4)
F1B—C14B—C15B—C16B	179.6 (3)	C13D—C14D—C15D—C16D	0.6 (7)
C14B—C15B—C16B—C11B	0.6 (6)	C14D—C15D—C16D—C11D	−1.5 (7)
C12B—C11B—C16B—C15B	−0.8 (6)	C12D—C11D—C16D—C15D	1.5 (7)
C10B—C11B—C16B—C15B	−179.3 (4)	C10D—C11D—C16D—C15D	−179.8 (4)

### Hydrogen-bond geometry (Å, º)

**Table:**

*D*—H···*A*	*D*—H	H···*A*	*D*···*A*	*D*—H···*A*
N1*A*—H1*A*···O1*B*^i^	0.86	2.01	2.868 (3)	173
N1*B*—H1*B*···O1*A*^i^	0.86	2.00	2.860 (3)	173
N1*C*—H1*C*···O1*D*^ii^	0.86	2.01	2.865 (3)	173
N1*D*—H1*D*···O1*C*^iii^	0.86	2.00	2.857 (4)	173

Symmetry codes: (i) −*x*+1, −*y*+1, −*z*+1; (ii) *x*−1, *y*−1, *z*−1; (iii) *x*+1, *y*+1, *z*+1.

## References

[bb1] Agilent (2012). *CrysAlis PRO* and *CrysAlis RED* Agilent Technologies, Yarnton, England.

[bb2] Allen, F. H., Kennard, O., Watson, D. G., Brammer, L., Orpen, A. G. & Taylor, R. (1987). *J. Chem. Soc. Perkin Trans. 2*, pp. S1–19.

[bb3] Aydogan, F., Ocal, N., Turgut, Z. & Yolacan, C. (2001). *Bull. Korean Chem. Soc.* **22**, 476–480.

[bb4] Fun, H.-K., Hemamalini, M., Sumangala, V., Nagaraja, G. K. & Poojary, B. (2011*b*). *Acta Cryst.* E**67**, o2835. 10.1107/S1600536811039845 PMC3247574 22219879

[bb5] Fun, H.-K., Hemamalini, M., Sumangala, V., Prasad, D. J. & Poojary, B. (2011*a*). *Acta Cryst.* E**67**, o2847. 10.1107/S1600536811039857 PMC3247585 22219890

[bb6] Fun, H.-K., Quah, C. K., Frank, P. V., Damodara, N. & Kalluraya, B. (2012). *Acta Cryst.* E**68**, o2192. 10.1107/S160053681202795X PMC3393992 22798857

[bb7] He, G.-F. & Shi, Z.-Q. (2011). *Acta Cryst.* E**67**, o48.

[bb8] Kundu, N., Chatterjee, P. B., Chaudhury, M. & Tiekink, E. R. T. (2005). *Acta Cryst.* E**61**, m1583–m1585.

[bb9] Macrae, C. F., Bruno, I. J., Chisholm, J. A., Edgington, P. R., McCabe, P., Pidcock, E., Rodriguez-Monge, L., Taylor, R., van de Streek, J. & Wood, P. A. (2008). *J. Appl. Cryst.* **41**, 466–470.

[bb10] Odabaşoğlu, M., Büyükgüngör, O., Sunil, K. & Narayana, B. (2007). *Acta Cryst.* E**63**, o4145–o4146.

[bb11] Sheldrick, G. M. (2008). *Acta Cryst.* A**64**, 112–122. 10.1107/S0108767307043930 18156677

[bb12] Taggi, A. E., Hafez, A. M., Wack, H., Young, B., Ferraris, D. & Lectka, T. (2002). *J. Am. Chem. Soc.* **124**, 6626–6635. 10.1021/ja0258226 12047183

[bb13] Xu, Z., Thompson, L. K. & Miller, D. O. (1997). *Inorg. Chem.* **36**, 3985–3995.

